# Effect of Training Load on Post-Exercise Cardiac Biomarkers in Healthy Children and Adolescents: A Systematic Review of the Existing Literature

**DOI:** 10.3390/jcm12062419

**Published:** 2023-03-21

**Authors:** Adamantia Papamichail, Emmanuel Androulakis, Andrew Xanthopoulos, Alexandros Briasoulis

**Affiliations:** 1 Department of Pediatrics, Larnaca General Hospital, Larnaca 6045, Cyprus; 2 Cardiology Department, St George’s University of London, Blackshaw Road, London SW17 0QT, UK; 3 Medical School of Athens, National and Kapodistrian University of Athens, Goudi, 11527 Athens, Greece

**Keywords:** cardiac troponin, cTn, running, treadmill, swimming, exercise, adolescents, children

## Abstract

Background: Postexercise release of cardiac biomarkers (cardiac troponins, cTn, and N-terminal pro b-type natriuretic peptide, NT-proBNP) is a well-known phenomenon in adults, although it remains unclear how it manifests in children. The aim of this review is to compare the pre-exercise with the post-exercise measurement of serum cardiac biomarkers, as well as to analyze their post-exercise release based on age, sex, and exercise intensity and duration. Methods: The terms troponin, football, swimmers, marathon, run, and exercise were used in a literature search at National Library of Medicine. The search was further refined by adding the keywords athletes, children, adolescents, and sport. Results: Fifteen pediatric studies and four studies with a mixed population of adults and children totaled 19 studies for the final analysis. In addition to them, some adult studies have been included for comparison. The kinetics of the cTn and NT-proBNP response after exercise have been the subject of our interest. While the impact of sport type, age, and sex has not yet been fully characterized, the existing data points to considerable impacts of sport intensity and duration on post-exercise biomarkers elevation. Most of the findings came from endurance sports, but the evidence is sparse. Furthermore, there is only limited data on women and less on young adults, African Caribbeans, and professional athletes. Conclusions: Both amateur and competitive athletes can exhibit post-exercise release of both cTn and NT-proBNP. This is transient and lacks pathological significance, in contrast with adult population, in which exercise-induced increases in in these biomarker levels may not always be benign. While NT-proBNP release is still primarily driven by activity duration, cTnT release is additionally affected by exercise intensity. To define individual ranges of normality for postexercise cTn and NT-proBNP elevation, the role of several confounders (age, sex, sport type/intensity etc.) remains to be further elucidated.

## 1. Introduction

Cardiac troponins T and I (cTnT and cTnI) are regarded as sensitive markers of myocardial injury (MI) and acute myocardial infarction (AMI) and are recognized indications of myocyte necrosis [1]. Following permanent cardiac muscle injury, serum levels of cTnT and cTnI rise and reach their peak during the following days [2,3]. The prohormone brain natriuretic peptide’s N-terminal fragment (NT-proBNP) is a marker that reflects myocardial stretch [4] and is currently used to diagnose heart failure and asymptomatic left ventricular dysfunction with the magnitude and duration of release dependent on the severity of stretch and stress [5,6,7].

Recent years have seen a significant reduction in the lower detection limits of cTnT and cTnI tests due to the availability of new, sensitive assays. These tests can detect cTn concentrations in at least 50% of a healthy population at rest and can identify the 99th percentile with a coefficient of variation of 10% [8]. Although these assays’ improved sensitivity allows for better true positive detection rates, a loss in specificity has been noted, raising the possibility that cTn presence may be linked to causes other than AMI. In this context, exercise is a recognized nonpathological source of cTn rise [2,9,10,11].

Numerous studies have documented the presence and the kinetics of the serological release of cTnT, cTnI, and NT-proBNP during exercise [12,13,14]. Most of the physiological processes that result in troponin release during exercise are unknown. It has been suggested that an increase in preload may contribute to an increase in myocardial stretch and the transfer of cTn molecules across the intact myocyte membranes by integrins. Posttranslational changes, such proteolytic degradation, phosphorylation, glycation, and acetylation, have an impact on the amount of circulating troponins. Individual variations in these alterations may modify the circulating cTn molecules and affect how well existing techniques detect cTn molecules [15]. Moreover, the hs-cTn release might be caused by a brief imbalance in oxygen uptake compared to oxygen supply, and the level of training may have an impact on hs-cTn elevation [16].

Contrary to an AMI-related release, cTn values typically peak between two and five (cTnT) and three to six (cTnI) hours after exercise, decline, and revert to baseline levels in most participants after 24 h of recovery. Following exercise, NT-proBNP release often achieves its peak and remains increased for the next 72 h [17,18,19]. Due to their shorter kinetics and lower Molecular Weight (MW) isoforms, hs-cTnI and hs-cTnT rise driven on by physical activity may be clearly separated from one another. It has been demonstrated that the major circulating forms following arduous exercise are degraded fragments (MW of 14–18 kDa) of cTnT. These lower molecular forms of cTnT appear to be more comparable to those seen in individuals with end-stage renal disease (ESRD) than in those with MI [16].

The decision threshold for the diagnosis of MI is set at the 99th percentile of a normal reference population, which is known as the upper reference limit (URL) for both general and pediatric populations. In this regard, the reported 99th percentiles for cTn and NT-proBNP, both of which are utilized for clinical diagnosis, are lower for children than for adults [11,20].

There are currently very few studies that have examined the cardiac biomarker response to exercise in children and adolescents. Furthermore, these studies have small sample numbers and uneven exercise exposure, which limits their statistical power. As a result, the relationship between cTn and NT-proBNP and exercise is still debatable and may be complicated by factors related to both the individual and the characteristics of exercise. Based on research involving adult participants, factors other than age may affect the release of cardiac biomarkers. It is unclear whether cTn and NT-proBNP show sex differences [19,21,22,23,24,25,26,27]. While training load may not be related to the emergence of biomarkers [28,29,30,31,32,33], prior exercise experience has been negatively connected with cTn release. Although its association with training load is still controversial, NT-proBNP is not related to previous exercise experience either. Finally, neither cTn nor NT-proBNP findings have been associated with fitness conditions [32,34,35,36,37,38]. The effects of exercise parameters on the release of cardiac biomarkers have also been investigated. Exercise duration has been related to both cTn and NT-proBNP data, whereas exercise intensity has been mentioned as a predictor for cTn release.

In contrast to the pediatric population, several studies have examined the kinetics of troponin levels in the adult population. All researchers agree that cTnI levels are related to baseline cTn concentration, exercise intensity, and exercise duration. A recent study has shown that an individual’s cTnI response, which is affected by the duration of high-intensity exercise and the timing of cTnI sampling, accounts for a considerable percentage of the variability in exercise-induced cTnI increase. Troponin levels are at their lowest prior to reaching their peaks at 3 h and 24 h, respectively. The greatest significant cTnI difference is occurring 3 h after the most demanding activity and the least demanding exercise. There are no variations in baseline echocardiographic parameters or race performance among participants with the highest or lowest cTnI levels [15].

Another study in adults showed that recreational runners have temporary ventricular shocking and transitory alterations in myocardial biomarkers after a 21 km run. One hour after exercise, all biomarkers rise, and by 24 h after the run, they return to their pre-exercise levels. The global longitudinal strain in the left ventricle and the free wall of the right ventricle (RV) are both reduced [39].

Young amateur athletes who participated in a 12-week exercise program have shown a substantial increase in cTnI levels, which has a positive correlation with systolic blood pressure (BP). Furthermore, there is statistically significant positive correlation between CTnI and mean arterial pressure. A strong and favorable connection between CTnI and resting heart rate was observed. Therefore, following 12-week adaptation to endurance training, serum CTnI is strongly and favorably correlated with cardiovascular parameters [40].

Given the modest cardiovascular risk that children and adolescents have, the levels of their cardiac biomarkers should be carefully examined. An analysis may be able to reveal potential relationships between personal traits and exercise levels that might partially account for the variety of the present findings. It is worth noticing that immature myocardium is more susceptible to injury than the myocardium in adult athletes [39].

This review’s major goal is to examine studies that involved healthy children and adolescents who exercised and whose resting and post-exercise measurements of cTnT, cTnI, and NT-proBNP are described. A secondary goal is to analyze the moderator effects of age, pubertal status, sex, previous training (years), current training (in hours per week or kilometers per week), exercise duration (in minutes), and exercise mode on the pooled effects determined by the main objective.

## 2. Methods

### 2.1. Search Strategy

We searched National Library of Medicine (PubMed) and Scholar databases. A 3-component additive search key (A AND B AND C) was used with: A, measurement; B, intervention; and C, population. The measurement was defined with the expression “cardiac biomarker” OR “Troponin” OR “TnT” OR “TnI” OR “cTn” OR “hs-cTn” OR “N-terminal prohormone of brain natriuretic peptide” OR “NT-proBNP” OR “NT-pro-BNP”. The intervention was specified with “exercise” OR “physical activity” OR “running” OR “marathon” OR “football” OR “swimming” OR “athletes”. Finally, the population was stated with “children” OR “adolescent” OR “young” OR “infants” OR “adults”.

### 2.2. Inclusion and Exclusion Criteria

We chose studies that used a repeated measures methodology (pre-exercise and post-exercise) and were either observational or experimental. The studies we included involved children and/or adolescents without underlying heart disease and with a normal resting electrocardiogram (ECG). Adult studies were included for comparison. The interventions that exposed participants to physical activity, such as sporting activities and laboratory investigations, were of interest. Blood samples were taken before exercise and at least once after exercise (immediately after, 1 and/or 2 and/or 3 and/or 4 h after). In addition, a measurement was taken 24 and 48 h after the exercise to determine the drop or not of the biomarkers, but also the peak of their increase. We searched primarily for studies that reported cTnT and/or cTnI and/or NT-proBNP concentrations before and after exercise. According to our exclusion criteria, we excluded the studies which reported children with any heart disease. Studies where participants were exposed to specific pharmacological or nutritional interventions were also excluded and the remaining articles were included in our review.

### 2.3. Data Extraction

Studies were inspected to gather the data for: sample size; sex; maturational status; age; training status (years of previous experience, weekly hours of training, and weekly kilometers of training); performed exercise; exposure duration (minutes); and absolute concentration of cTnT, cTnI, or NT-proBNP before and after exercise. All concentrations were expressed in nanograms per liter (ng/L) [1].

## 3. Results

The inclusion/exclusion criteria were met by 19 studies, with a total population of 478 participants (mean sample size of 25.3 participants) and a mean age of 14.8 years (± standard deviation, 2.1). Interventions were based on nine distinct modalities: in one investigation (children population), participants ran a full-marathon (42 km); in three (children population), they ran a half-marathon (21 km); in seven (one with mixed and six with children population), they had a treadmill or outdoor run in distance non related to a marathon; in one (children population) they played basketball; in one (mixed population) they played football; in one (children population), they played soccer; in three (two with mixed and one with children population) they swam; in one (children population), they had cycling; and one (children population) included a set of table tennis exercises (Table 1 and Table 2).

### 3.1. Cardiac Troponin T

At baseline, participants’ average cTnT concentration was 5 ng/L (4–6 ng/L). After 2 to 5 h, this concentration increased and peaked at 144 ng/L (83–205 ng/L). cTnT was reduced with a pooled concentration of 11 ng/L (5–16 ng/L), which was somewhat higher than at baseline, 24 h after exercise. This time frame of decreasing troponin’s level has been also shown in adults [56], in whom troponin kinetics have the same distribution. Nevertheless, the absolute values of cTnT (as well as cTnI) are higher in the adult population. The three pooled concentrations were all diverse between studies, as were their differences. A cTnT peak was observed in 69% of subjects above the URL. For comparison, it is worth mentioning that elevated troponin levels in studies with adults have been observed at a percentage of participants up to 75% [57]. Eight different activity types—a full-marathon, a half-marathon, a treadmill running, a football-7 game, a soccer game, table tennis, basketball, and swimming—were examined in the subgroups analysis for cTnT. A post hoc study revealed that cTnT rises were higher after a full or half marathon and a treadmill run than after intermittent table tennis and swimming. It is important to note that cTnT rise was positively correlated with intensity and duration and negatively correlated with age. There is no association between the rise in cTnT and gender, Tanner stage, years of prior training, or weekly training kilometers.

### 3.2. Cardiac Troponins I

The pooled baseline value for cTnI was 16 ng/L (10–22 ng/L). Participants increased this concentration up to a high of 248 ng/L (17–478 ng/L) after 3 to 6 h of exercise exposure. This dropped to 38 ng/L (19–56 ng/L) after a 24-h recovery period, which was statistically identical to the projected peak concentration. All three of the pooled concentrations, together with their variations, were diverse between trials, nevertheless, 47% of individuals had their cTnI higher than the URL. The corresponding percentage of adults in the relevant studies is up to 93%. Six different activity types—a full-marathon, a half-marathon, treadmill running, basketball, table tennis, and football—were used to quantify cTnI for the subgroup analysis. There was no difference in the peak cTnI rise between exercise types, age groups, intensities, or durations. Furthermore, we could not discover any differences brought on by years of training.

### 3.3. N-Terminal Prohormone Brain Natriuretic Peptide

NT-proBNP had a pooled baseline value of 77 ng/L (14–140 ng/L). Directly following exercise, this concentration increased, achieving a peak of 106 ng/L (17–195 ng/L). Finally, it did not vary from its peak or baseline 24 h after exercise, with an estimate of 83 ng/L (0–182 ng/L). In total, 13% of subjects had NT-proBNP concentrations that were higher than the URL. This proportion of participants is much lower than in adults; up to 67% of adult participants in adult studies exceeded the URL [58]. In the subgroup analysis, four distinct activity modes—a half marathon, treadmill running, swimming, and football—showed the presence of NT-proBNP. The primary factor affecting the post-exercise rise in NT-proBNP was exercise modality. Post hoc comparisons showed that the larger NT-proBNP increases were associated with swimming being followed by half and then by the latter. In addition, the duration of exercise was positively correlated with the estimate whereas age and intensity were not. Finally, there are no variations in NT-proBNP for sex, Tanner stage, or years of prior training.

## 4. Discussion

This review’s major goal is to determine how exercise influenced the levels of cTnT, cTnI, and NT-proBNP in children’s and adolescents’ plasma. Overall, we discovered that: (1) all three biomarkers were significantly increased after exercise; (2) only cTnT showed a significant decline from peak values after a 24-h recovery; (3) individual variability was seen between studies; and (4) exercise duration affected both cTnT and NT-proBNP while intensity only affected cTnT. Despite these conclusions, the analysis for publication bias and the quality evaluation of research showed that current studies had a reasonable degree of quality.

### 4.1. Cardiac Troponins T and I

Our findings suggest that physical activity causes cTn release in children and adolescents. Data indicate a rapid spike in cTnT within the first hours after recovery and a practically full return to baseline at 24 h. Although statistical power was constrained and resulted in only marginally significant differences between peak and 24-h values, similar outcomes were discernible for cTnI. These findings imply that cTn variations in children and adolescents over the period of a 24-h recovery are equivalent to those observed in adults [12,59]. Our findings are consistent with other studies that found the maximum cTnT and cTnI concentrations occurred between two and three and three and five hours after exercise, respectively. According to the existing data, exercise-induced cTn release is subject to significant individual variation, much like in the case of adults. Controlled research with adolescents and adults has shown that exercise volume influences cardiac work and that the relationship between exercise volume and cTnT variability is also partially explained. The proportion of people surpassing the URL in our study is larger than that reported by a recent meta-analysis without age limitations, which may be due to the increased cTnT release we saw in the younger participants. Some of the adolescents included in the study exceeded the reference levels for an acute myocardial infarction, which recovered within 24 h. Previous authors suggested that the less mature myocardium in younger people may be more sensitive to exercise-induced increases in cTn. This would indicate that maturity is involved in modulating the release of cTn following the exercise. Furthermore, based on the available data, no correlation between cTnT release and the puberty phase was found, even though the puberty phase positively affects absolute cTnT (but not its elevation). Furthermore, we could not discover any correlation between cTnT release and pubertal state using the scant data currently available. Running appears to cause more cTnT releases than other types of sport, while it is difficult to confirm this claim through side-by-side comparisons [60]. We were unable to confirm whether the cTnT rise is caused by recent or past training. Whether there were any sex differences in the cTn release was not immediately apparent. This is consistent with adult research that found gender and training history had no effect on cTn release. The limited number of trials made it impossible to account for the between-subject heterogeneity in cTnI release.

### 4.2. N-Terminal Prohormone Brain Natriuretic Peptide

Without a substantial decrease throughout the 24-h recovery period, a rise in NT-proBNP immediately following exercise was confirmed, supporting earlier findings with adults [31,61]. NT-proBNP could have a clearance time that is longer than cTn, possibly reaching 72 h [22,62]. This might be an early cardiac response to the demanding training stimulus in children and adolescents [63]. We noticed that exercise-induced NT-proBNP alterations were less pronounced than those shown in cTn. These variations might be influenced by age. However, no changes in NT-proBNP for age and pubertal status were discovered in either our research [19,55] or earlier studies that explicitly compared adolescents with adults. Therefore, it is reasonable to assume that these discrepancies may be explained by shorter exercises in studies with adolescents compared to their equivalents with adults. Our findings support recent reports from research with adults showing the duration of exercise has a significant impact on the release of NT-proBNP in adolescents [32,64]. Given the strong correlation between pre-exercise and postexercise results [65], baseline variations between trials may contribute to the variations in NT-proBNP peak levels we observed depending on the kind of exercise. The production of NT-proBNP in response to exercise is largely unaffected by factors such as exercise intensity, training, fitness, and sex, as was also validated by our findings in adults.

### 4.3. Clinical Implications

Despite some cross heterogeneity, most patients in all included studies found a release of cardiac biomarkers. Importantly, our data demonstrate that the mechanisms controlling cardiac biomarkers after exercise as well as their kinetics in children and adolescents are like those found in earlier investigations in adults. This, according to some studies, is a reversible cellular process that is brought on by a typical physiological response to exercise [34,66,67,68]. Similarly, the rise in cTn may be due to an increase in the rate and power of cardiac contraction during exercise, which results in temporary membrane disruption and makes it possible for cytosolic cTn to enter the bloodstream [69]. However, the release of NT-proBNP by the ventricular cardiomyocytes during exercise may indicate a volume overload and stretch of the cardiac wall [62]. In this context, our review extends this to children and adolescents and recommends that thorough information regarding any recent exercise should be collected when considering cTnT, cTnI, and NT-proBNP in emergency circumstances [70].

### 4.4. Limitations

In the existing published studies, there are several weaknesses. First of all, the population samples they have relied on are mostly very small (8–70 cases per study), and as a result, their conclusions are not reproducible in a wider part of the population, nor do they have great statistical power. Another limitation of this review is the insufficient data presented by a variety of different research. Some nonsignificant results could be explained by this lack of statistical power. In addition, there is heterogeneity in the studied variables, as well as in the time frame of their measurement. The methods of measuring cardiac troponins (cTnT and cTnI) differ between the included studies in terms of detection the sensitivity (some studies had measured the high sensitivity types while others had measure the basic form) of them, as well as the antibodies used. It is extremely challenging to address the pathophysiological significance of elevated cTn levels following vigorous exercise in children in accordance with the diagnosis of cardiac damage, as required by international guidelines. Throughout neonatal age to the age of 18, there are no accurate statistics on the precise estimation of the 99th percentile URL. Additionally, several studies used a mixed sample (both adults and children) of the population but did not draw distinct, in-depth results.

## 5. Conclusions

Children’s and adolescents’ cardiac biomarkers seem to elevate after aerobic exercise; a finding which is consistent with the corresponding findings for adults. Of course, this phenomenon is transient and lacks pathological significance, in contrast with adult population, in which exercise-induced increases in hs-cTn may not always be benign. Additionally, while NT-proBNP release is still primarily driven by activity duration, cTnT release is additionally affected by exercise intensity. Adolescent heart seems to cope well with two high-duration sports performed on the same day. Overall, to define individual ranges of normality for postexercise cTn elevation, the role of several confounders (age, sex, sport type/intensity/duration, and training level) remains to be further elucidated.

## Figures and Tables

**Table 1:** Studies reflecting on effects of exercise on cardiac biomarkers in pediatric populations.

Author	Type of Exercise	Population	Parameters Tested	Conclusions
Pompa et al., 2022 [41]	Treadmill exercise test	13 children	ECG, cTnT prior to exercise and 4 h after	Troponin T levels did not increase as a result of exercise; if they were found elevated, this meant that there is an underlying heart disease
Tong et al., 2021 [42]	7–21 km treadmill run	12 male adolescents	ECG (and other tests non related to the cardiac function)	Running did not cause any ECG abnormalities
Cirer-Sastre et al., 2021 [17]	30 min, high-intensity swimming	70 male children 7–18 yo	cTnT (before, immediately after, and 3 h after exercise)	Baseline cTnT levels are directly related to the Tanner stage However, this elevation does not depend on maturational status
Cirer-Sastre et al., 2019 [43]	Soccer	20 children (11.9 ± 2 yo)	hs-cTnT (before and 3 h after exercise)	As time (since the beginning of the exercise) passes, the proportion of children who exceed the troponin threshold levels for MI increases
Peretti et al., 2018 [44]	Cycling	21 male preadolescent athletes (age 9.2 ± 1.7 yrs)	hs-cTnT, ΝΤ-proBNP, CK-MB, CK, ECG-Holter, heart rate	Some athletes showed an elevation in their troponin, CK, and NT-proBNP levels. Those with elevated troponin were less trained than those with normal values. CK levels elevation is positively related to the duration of exercise
Kong et al., 2017 [18]	6–21 km run (6–7 times/week)	19 male children (16.1 ± 1.2 yo) 19 girls (15.9 ± 1.4)	cTnT (pre-exercise and 4 h post-exercise), echocardiogram	Males had greater troponin levels than females Estrogen probably has a protective role against troponin elevation Athletes’ normal troponin ranges should not be the same as the general pediatric population
Ma et al., 2014 [45]	Table tennis (2 h/day, 5 times a week)	28 male children (7.21 ± 1.11 yo)	cTnT and cTnI and CK-MB (before exercise, immediately after, 4 h, 24 h, and 48 h postexercise) echocardiogram	The levels of all enzymes were elevated immediately after exercise. Troponin levels returned to baseline 48 h after exercise. In some individuals, these levels exceeded the cut-off for MI, while in others, CK-MB levels did not return to normal 24 h after exercise
Traiperm et al., 2012 [46]	Marathon (42 km run)	40 adolescents (20 females) 13–17 years old	cTnT and cTnI (before, immediately after and 24 h post-exercise)	Most of the participants noted an elevation in all biomarker levels, but these were normal 24 h post-exercise. Cardiac biomarkers elevation is a result of exercise, but an underlying cardiac disease cannot be completely ruled out
Nie et al., 2011 [47]	21 km run	63 adolescent runners 16.4 ± 1.5 yo 10 females	cTnT (pre-exercise and 24 h postexercise) ECG, echocardiogram	Cardiac biomarkers elevation was inversely related to age and training level
Nie et al., 2011 [48]	2 bouts of prolonged exercise run	12 male adolescent runners 14.5 ± 1.5 yo	cTnT and NT-pro-BNP (before, immediately after, and 255 min after each bout), echocardiogram	LV ejection fraction decreased after the 1st bout of run (but remained within normal range). The E:A ratio was found to have a higher reduction after the 1st bout than after the 2nd. Systolic blood pressure and LV meridional wall stress increased significantly after each round
Nie et al., 2011 [49]	21 km run	12 male adolescents 16.2 ± 0.6 yo	cTnT, cTnI, CK-MB, CK, LDH (all at rest, 2, 4, and 24 h after exercise), echocardiogram, ECG	The levels of all biomarkers were elevated. Some of the participants had values above the cut-off for MI. Nevertheless, their rapid return to normal range indicates that the damage is transient and not pathological
Fu et al., 2009 [36]	Treadmill exercise	13 adolescent runners 14.8 ± 1.6 yo	cTnT (immediately after and 5 h after exercise), ECG, echocardiogram	The greater elevation of troponin levels was found 5 h after the exercise
Nie et al., 2007 [50]	Basketball	10 male adolescent players 15.0 ± 0.7 yo	cTnT and cTnI (immediately before, and at 2, 4, and 24 h post-exercise), ECG, echocardiogram	Within 24 h of exercise, troponin levels return to normal range, indicating that elevation in troponin levels is not due to pathology, but due to transient exercise-induced changes in the heart
Tian et al., 2006 [51]	21 km run (treadmill)	10 trained male adolescents 16.2 ± 0.6 yo	cTnI and cTnT (immediately before and 2, 4, and 24 h after exercise), VO_2_, running speed	Serum cTnT and cTnI in some individuals (4 h after the run) were above the cut-off for MI, but returned towards pre-exercise levels within 24 h. The elevation in troponin levels is inversely related to the training level (those with increased troponin levels were of lower training status), VO_2_ and running speed, and positively related to their personal best half- and marathon races
Chuang et al., 1995 [52]	Running group A: 5 km/day group B: 7 km/day	24 children 16–17 yo	RR, BP, PR, body surface skin temperature (at rest and after exercise), cTnT CK, CK-MB, LDH	After one week, the CK levels in both groups were higher after exercise; in group A, they decreased to the normal values and stayed the same throughout the fifth week, while in group B, they decreased to the normal value after training for three weeks, and there were significant differences between the values before exercising and those for the fourth and fifth weeks of training. Although the levels of CK-MB, LDH, and Troponin-T increased after training, all were within the normal range, and no significant difference was observed before or after five weeks of training

Abbreviations: cTnI: cardiac troponin I, cTnT: cardiac troponin T; CK: Creatin kinase; CK-MB: Creatine kinase -MB; LDH: lactate dehydrogenase; RR: respiratory rate; BP: blood pressure; PR: pulse rate; NT-pro-BNP: NT-pro-brain natriuretic peptide; ECG: electrocardiogram; MI: myocardial injury; LV: left ventricle.

**Table 2:** Studies reflecting on effects of exercise on cardiac biomarkers in mixed paediatric/adult populations.

Author	Type of Exercise	Population	Parameters Tested	Conclusions
Cirer-Sastre et al., 2021 [53]	Swimming	32 trained males (18 adolescents, 14 ± 3 yo and 14 adults, 35 ± 9 yo)	ECG, cTnT (before, immediately, and 3 h after exercise)	cTnT levels increase in all athletes, but they found to be unrelated to the exercise intensity. In the pediatric population, the rise is greater and occurs later (peak values are the same for both populations)
Cirer-Sastre et al., 2020 [54]	Football 7 match	24 adolescents (10.7 ± 1.6 yo)	hs-cTnT (before, and 3 h after exercise), ECG	Children’s resting levels of cTnT were lower than those of adults. 3 h after the game, both groups had increased cTnT. Nevertheless, the absolute post-match concentration and the rise were equal among groups. At baseline, none of the participants went over the URL. However, at 3 h after activity, 66.67% of adults and 70.83% of children surpassed the threshold for MI
Legaz-Arres et al., 2017 [19]	Swimming	50 Adolescent swimmers, 12–18 yo and 16 adults, 22–46 years old	ECG, hs-cTnT and NT-proBNP (before, immediately after and at 1, 3, 6, 12, and 24 h post-exercise)	Males have higher baseline troponin T levels and greater increases in them (in contrast to NT-proBNP which is not sex-dependent)
Tian et al., 2012 [55]	90-min treadmill exercise	13 male adolescents (14.1 ± 1.1 years old) and 13 male adults (24.0 ± 3.6 years old)	hs-cTnT and NT-pro-BNP (pre-exercise, immediately after, and 1, 2, 3, 4, 5 6, and 24 h post-exercise), echocardiogram (pre-exercise, immediately after and 6 h post-exercise)	Troponin levels were elevated in all participants. Peak levels in Tanner 2 stage was higher compared to those in Tanner 3 (but not statistically significant). Markers of systolic function were reduced, cardiac output and heart rate were both increased. Athletes’ normal biomarker ranges should be different from the general population

Abbreviations: cTnI: cardiac troponin I, cTnT: cardiac troponin T; NT-pro-BNP: NT-pro-brain natriuretic peptide; ECG: electrocardiogram; MI: myocardial injury; LV: left ventricle.

## References

[1] Thygesen K., Alpert J.S., Jaffe A.S., Simoons M.L., Chaitman B.R., White H.D. (2012). Third Universal Definition of Myocardial Infarction. Circulation.

[2] Alquézar Arbé A., Santaló Bel M., Sionis A. (2015). Clinical interpretation of high sensitivity troponin T. Med. Clín..

[3] Sandoval Y., Smith S.W., Love S.A., Sexter A., Schulz K., Apple F.S. (2017). Single High-Sensitivity Cardiac Troponin I to Rule Out Acute Myocardial Infarction. Am. J. Med..

[4] Thomas M., Lip G.Y. (2017). Novel Risk Markers and Risk Assessments for Cardiovascular Disease. Circ. Res..

[5] Bayés-Genís A. (2005). NTproBNP circulante, un nuevo biomarcador para el diagnóstico del paciente con disnea aguda. Rev. Esp. Cardiol..

[6] Gaggin H.K., Januzzi J.L. (2013). Biomarkers and diagnostics in heart failure. Biochim. Biophys. Acta.

[7] Panagopoulou V., Deftereos S., Kossyvakis C., Raisakis K., Giannopoulos G., Bouras G., Pyrgakis V., Cleman M.W. (2013). NTproBNP: An Important Biomarker in Cardiac Diseases. Curr. Top. Med. Chem..

[8] Sandoval Y., Smith S.W., Apple F.S. (2016). Present and Future of Cardiac Troponin in Clinical Practice: A Paradigm Shift to High-Sensitivity Assays. Am. J. Med..

[9] Haider D.G., Klemenz T., Fiedler G.M., Nakas C., Exadaktylos A.K., Leichtle A.B. (2017). High sensitive cardiac troponin T: Testing the test. Int. J. Cardiol..

[10] Meigher S., Thode H.C., Peacock W.F., Bock J.L., Gruberg L., Singer A.J. (2016). Causes of Elevated Cardiac Troponins in the Emergency Department and Their Associated Mortality. Acad. Emerg. Med..

[11] Cirer-Sastre R., Legaz-Arrese A., Corbi F., George K., Nie J., Carranza-García L.E., Reverter-Masià J. (2019). Cardiac Biomarker Release After Exercise in Healthy Children and Adolescents: A Systematic Review and Meta-Analysis. Pediatr. Exerc. Sci..

[12] Gresslien T., Agewall S. (2016). Troponin and exercise. Int. J. Cardiol..

[13] Jarolim P., Morrow D.A. (2016). Use of high sensitivity cardiac troponin assays as an adjunct to cardiac stress testing. Clin. Biochem..

[14] Scharhag J., George K., Shave R., Urhausen A., Kindermann W. (2008). Exercise-Associated Increases in Cardiac Biomarkers. Med. Sci. Sports Exerc..

[15] Bjørkavoll-Bergseth M., Erevik C.B., Kleiven Ø., Eijsvogels T.M.H., Skadberg Ø., Frøysa V., Wiktorski T., Auestad B., Edvardsen T., Aakre K.M. (2021). Determinants of Interindividual Variation in Exercise-Induced Cardiac Troponin I Levels. J. Am. Heart Assoc..

[16] Clerico A., Aimo A., Cantinotti M. (2021). High-sensitivity cardiac troponins in pediatric population. Clin. Chem. Lab. Med..

[17] Cirer-Sastre R., Legaz-Arrese A., Corbi F., López-Laval I., George K., Reverter-Masia J. (2021). Influence of maturational status in the exercise-induced release of cardiac troponin T in healthy young swimmers. J. Sci. Med. Sport.

[18] Kong Z., Nie J., Lin H., George K., Zhao G., Zhang H., Tong T.K., Shi Q. (2016). Sex differences in release of cardiac troponin T after endurance exercise. Biomarkers.

[19] Legaz-Arrese A., Carranza-García L.E., Navarro-Orocio R., Valadez-Lira A., Mayolas-Pi C., Munguía-Izquierdo D., Reverter-Masía J., George K. (2017). Cardiac Biomarker Release after Endurance Exercise in Male and Female Adults and Adolescents. J. Pediatr..

[20] Cantinotti M., Clerico A., Giordano R., Assanta N., Franchi E., Koestenberger M., Marchese P., Storti S., D’Ascenzi F. (2021). Cardiac Troponin-T Release After Sport and Differences by Age, Sex, Training Type, Volume, and Intensity: A Critical Review. Clin. J. Sport Med..

[21] Carranza-García L.E., George K., Serrano-Ostáriz E., Casado-Arroyo R., Caballero-Navarro A.L., Legaz-Arrese A. (2011). Cardiac Biomarker Response to Intermittent Exercise Bouts. Int. J. Sports Med..

[22] Eijsvogels T., George K., Shave R., Gaze D., Levine B.D., Hopman M.T., Thijssen D.H. (2010). Effect of Prolonged Walking on Cardiac Troponin Levels. Am. J. Cardiol..

[23] Fortescue E.B., Shin A.Y., Greenes D.S., Mannix R.C., Agarwal S., Feldman B.J., Shah M.I., Rifai N., Landzberg M.J., Newburger J.W. (2007). Cardiac Troponin Increases Among Runners in the Boston Marathon. Ann. Emerg. Med..

[24] Jassal D.S., Moffat D., Krahn J., Ahmadie R., Fang T., Eschun G., Sharma S. (2009). Cardiac Injury Markers in Non-elite Marathon Runners. Int. J. Sports Med..

[25] Lippi G., Schena F., Dipalo M., Montagnana M., Salvagno G.L., Aloe R., Guidi G.C. (2012). Troponin I measured with a high sensitivity immunoassay is significantly increased after a half marathon run. Scand. J. Clin. Lab. Investig..

[26] Roca E., Nescolarde L., Lupón J., Barallat J., Januzzi J.L., Liu P., Pastor M.C., Bayes-Genis A. (2017). The Dynamics of Cardiovascular Biomarkers in non-Elite Marathon Runners. J. Cardiovasc. Transl. Res..

[27] Vidotto C., Tschan H., Atamaniuk J., Pokan R., Bachl N., Müller M.M. (2005). Responses of N-Terminal Pro-Brain Natriuretic Peptide (NT-proBNP) and Cardiac Troponin I (cTnI) to Competitive Endurance Exercise in Recreational Athletes. Int. J. Sport. Med..

[28] Hewing B., Schattke S., Spethmann S., Sanad W., Schroeckh S., Schimke I., Halleck F., Peters H., Brechtel L., Lock J. (2015). Cardiac and renal function in a large cohort of amateur marathon runners. Cardiovasc. Ultrasound.

[29] Hubble K.M., Fatovich D.M., Grasko J.M., Vasikaran S.D. (2009). Cardiac troponin increases among marathon runners in the Perth Marathon: The Troponin in Marathons (TRIM) study. Med. J. Aust..

[30] Neubauer O., König D., Kern N., Nics L., Wagner K.-H. (2008). No Indications of Persistent Oxidative Stress in Response to an Ironman Triathlon. Med. Sci. Sports Exerc..

[31] Legaz-Arrese A., López-Laval I., George K., Puente-Lanzarote J.J., Moliner-Urdiales D., Ayala-Tajuelo V.J., Mayolas-Pi C., Reverter-Masia J. (2015). Individual variability in cardiac biomarker release after 30 min of high-intensity rowing in elite and amateur athletes. Appl. Physiol. Nutr. Metab..

[32] Serrano-Ostáriz E., Terreros-Blanco J.L., Arrese A.L., George K., Shave R., Bocos-Terraz P., Izquierdo-Álvarez S., Bancalero J.L., Echavarri J.M., Quílez J. (2011). The impact of exercise duration and intensity on the release of cardiac biomarkers. Scand. J. Med. Sci. Sports.

[33] Urhausen A., Scharhag J., Herrmann M., Kindermann W. (2004). Clinical significance of increased cardiac troponins T and I in participants of ultra-endurance events. Am. J. Cardiol..

[34] Eijsvogels T.M.H., Fernandez A.B., Thompson P.D. (2016). Are There Deleterious Cardiac Effects of Acute and Chronic Endurance Exercise?. Physiol. Rev..

[35] Eijsvogels T.M., Hoogerwerf M.D., Oudegeest-Sander M.H., Hopman M.T., Thijssen D.H. (2014). The impact of exercise intensity on cardiac troponin I release. Int. J. Cardiol..

[36] Fu F., Nie J., Tong T. (2009). Serum Cardiac Troponin T in Adolescent Runners: Effects of Exercise Intensity and Duration. Int. J. Sport. Med..

[37] Scharhag J., Herrmann M., Urhausen A., Haschke M., Herrmann W., Kindermann W. (2005). Independent elevations of N-terminal pro–brain natriuretic peptide and cardiac troponins in endurance athletes after prolonged strenuous exercise. Am. Heart J..

[38] Voets P.J.G.M., Maas R.P.P.W.M. (2016). Serum cardiac troponin I analysis to determine the excessiveness of exercise intensity: A novel equation. J. Theor. Biol..

[39] Yeo T.J., Ling L.H., Lam C.S.P., Chong J.P.C., Liew O.W., Teo Z.L., Gong L., Richards A.M., Chan M.Y. (2020). Cardiac and renal biomarkers in recreational runners following a 21 km treadmill run. Clin. Cardiol..

[40] Tesema G., George M. (2021). Associations between cardiac troponin I and cardiovascular parameters after 12-week endurance training in young moderately trained amateur athletes. BMJ Open Sport Exerc. Med..

[41] Pompa A.G., Arora G., Harris T.H. (2022). Effect of treadmill exercise stress testing on troponin levels in children and adolescents. Cardiol. Young.

[42] Tong T., Baker J., Henriquez F., Shi Q., Zhang H., Kong Z., Nie J. (2021). A Combined Approach for Health Assessment in Adolescent Endurance Runners. Healthcare.

[43] Cirer-Sastre R., Legaz-Arrese A., Corbi F., López-Laval I., Puente-Lanzarote J., Hernández-González V., Reverter-Masià J. (2019). Effect of Training Load on Post-Exercise Cardiac Troponin T Elevations in Young Soccer Players. Int. J. Environ. Res. Public Health.

[44] Peretti A., Mauri L., Masarin A., Annoni G., Corato A., Maloberti A., Giannattasio C., Vignati G. (2017). Cardiac Biomarkers Release in Preadolescent Athletes After an High Intensity Exercise. High Blood Press. Cardiovasc. Prev..

[45] Ma G., Liu Y., Liu K. (2014). Influence of Repeated Bouts of Table Tennis Training on Cardiac Biomarkers in Children. Pediatr. Cardiol..

[46] Traiperm N., Gatterer H., Wille M., Burtscher M. (2012). Cardiac troponins in young marathon runners. Am. J. Cardiol..

[47] Nie J., George K.P., Tong T.K., Gaze D., Tian Y., Lin H., Shi Q. (2011). The Influence of a Half-Marathon Race Upon Cardiac Troponin T Release in Adolescent Runners. Curr. Med. Chem..

[48] Nie J., George K.P., Tong T.K., Tian Y., Shi Q. (2011). Effect of Repeated Endurance Runs on Cardiac Biomarkers and Function in Adolescents. Med. Sci. Sport. Exerc..

[49] Nie J., Tong T.K., George K., Fu F.H., Lin H., Shi Q. (2010). Resting and post-exercise serum biomarkers of cardiac and skeletal muscle damage in adolescent runners. Scand. J. Med. Sci. Sport..

[50] Nie J., Tong T., Shi Q., Lin H., Zhao J., Tian Y. (2008). Serum Cardiac Troponin Response in Adolescents Playing Basketball. Int. J. Sport. Med..

[51] Tian Y., Nie J., Tong T.K., Cao J., Gao Q., Man J., Shi Q., Liu W. (2006). Changes in serum cardiac troponins following a 21-km run in junior male runners. J. Sport. Med. Phys. Fit..

[52] Chuang C.C., Lee S.Y., Wang K.T. (1995). Impact of exercise on athlete’s cardiac muscle evaluated by Troponin T test. Gaoxiong Yi Xue Ke Xue Za Zhi Kaohsiung J. Med. Sci..

[53] Cirer-Sastre R., Corbi F., López-Laval I., Carranza-García L.E., Reverter-Masià J. (2021). Exercise-Induced Release of Cardiac Troponins in Adolescent vs. Adult Swimmers. Int. J. Environ. Res. Public Health.

[54] Cirer-Sastre R., Legaz-Arrese A., Corbi F., López-Laval I., Puente-Lanzarote J.J., Hernández-González V., Reverter-Masia J. (2020). Cardiac Troponin T Release after Football 7 in Healthy Children and Adults. Int. J. Environ. Res. Public Health.

[55] Tian Y., Nie J., Huang C., George K.P. (2012). The kinetics of highly sensitive cardiac troponin T release after prolonged treadmill exercise in adolescent and adult athletes. J. Appl. Physiol..

[56] Özgünen K., Günaştı Ö., Özdemir Ç., Korkmaz Eryılmaz S., Gezgin E., Boyraz C., Kılcı A., Adaş Ü., Kurdak S.S. (2022). The relationship between cardiac damage biomarkers and heart rate variability following 60 min of running. Clin. Auton. Res. Off. J. Clin. Auton. Res. Soc..

[57] Cirer-Sastre R., Jiménez-Gaytán R., Carranza-García L.E., George K., Apple F.S., Navarro-Orocio R., López-García R., Reverter-Masía J., Mayolas-Pi C., Morales-Corral P.G. (2022). A comparison of modelled serum cTnT and cTnI kinetics after 60 min swimming. Biomarkers.

[58] Kosowski M., Młynarska K., Chmura J., Kustrzycka-Kratochwil D., Sukiennik-Kujawa M., Todd J.A., Jankowska E.A., Banasiak W., Reczuch K., Ponikowski P. (2018). Cardiovascular stress biomarker assessment of middle-aged non-athlete marathon runners. Eur. J. Prev. Cardiol..

[59] Klinkenberg L.J., Luyten P., van der Linden N., Urgel K., Snijders D.P., Knackstedt C., Dennert R., Kietselaer B.L., Mingels A.M., Cardinaels E.P. (2016). Cardiac Troponin T and I Release After a 30-km Run. Am. J. Cardiol..

[60] Shave R., George K.P., Atkinson G., Hart E., Middleton N., Whyte G., Gaze D., Collinson P.O. (2007). Exercise-Induced Cardiac Troponin T Release. Med. Sci. Sport. Exerc..

[61] Lippi G., Schena F., Montagnana M., Salvagno G.L., Guidi G.C. (2008). Influence of acute physical exercise on emerging muscular biomarkers. Clin. Chem. Lab. Med..

[62] Katz R.K., Marshall D.B., Romanowski S.B., Stewart N.F. (1985). Katz et al. Reply. Soc. Work.

[63] Socrates T., Arenja N., Mueller C. (2009). B-Type Natriuretic Peptide in Children. J. Am. Coll. Cardiol..

[64] Serrano-Ostáriz E., Legaz-Arrese A., Terreros-Blanco J.L., López-Ramón M., Cremades-Arroyos D., Álvarez-Izquierdo S., Boscos-Terraz P. (2009). Cardiac Biomarkers and Exercise Duration and Intensity During a Cycle-Touring Event. Clin. J. Sport Med..

[65] Legaz-Arrese A., López-Laval I., George K.P., Puente-Lanzarote J.J., Pi C.M., Serrano-Ostáriz E., Revilla-Martí P., Moliner-Urdiales D., Reverter-Masia J. (2015). Impact of an endurance training program on exercise-induced cardiac biomarker release. Am. J. Physiol. Circ. Physiol..

[66] Nie J., George K., Duan F., Tong T.K., Tian Y. (2016). Histological evidence for reversible cardiomyocyte changes and serum cardiac troponin T elevation after exercise in rats. Physiol. Rep..

[67] Sanchis-Gomar F., Joyner M.J., Lucia A. (2015). Letter by Sanchis-Gomar et al Regarding Article, Cardiac Remodeling in Response to 1 Year of Intensive Endurance Training. Circulation.

[68] Herrmann M., Scharhag J., Miclea M., Urhausen A., Herrmann W., Kindermann W. (2003). Post-Race Kinetics of Cardiac Troponin T and I and N-Terminal Pro-Brain Natriuretic Peptide in Marathon Runners. Clin. Chem..

[69] Shave R., George K., Gaze D. (2007). The influence of exercise upon cardiac biomarkers: A practical guide for clinicians and scientists. Curr. Med. Chem..

[70] López-Laval I., Legaz-Arrese A., George K., Serveto-Galindo O., González-Rave J.M., Reverter-Masia J., Munguía-Izquierdo D. (2016). Cardiac troponin I release after a basketball match in elite, amateur and junior players. Clin. Chem. Lab. Med..

